# Supplementary material for: Alu-dependent RNA editing of GLI1 promotes malignant regeneration in multiple myeloma
Source: Nat Commun. 2017 Dec 4;8:1922. doi: 10.1038/s41467-017-01890-w (PMC5715072; doi:10.1038/s41467-017-01890-w)
Supplement: Supplementary file 1 — Supplementary Information [file 41467_2017_1890_MOESM1_ESM.pdf]

Supplementary Figure 1, Related to Figure 1

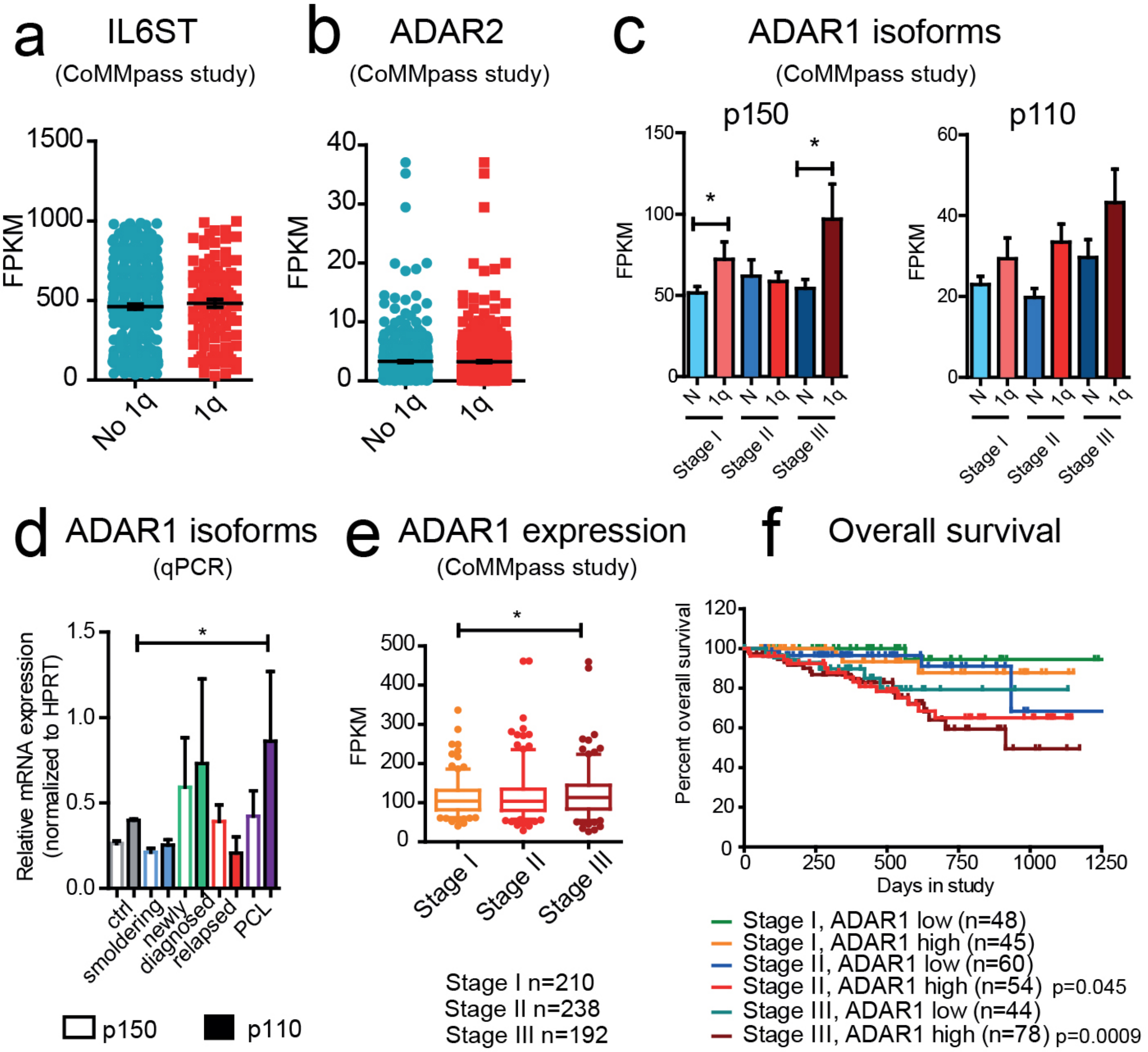

Supplementary Figure 1: (a) IL-6ST relative expression data in FPKM from CoMMpass study RNA-Seq data, from patients harboring 1q amplifications, compared to no 1q. The error bars represent ± S.E.M. of the mean. (b) ADAR2 relative expression data in FPKM from CoMMpass study RNA-Seq data, from patients harboring 1q amplifications, compared to no 1q. The error bars represent ± S.E.M. of the mean. (c) ADAR1 isoform relative expression in 1q-amplified (1q) versus no 1q patients. Error bars represent means ± S.E.M.; *p<0.05 by two-tailed, Mann-Whitney U test. (d) ADAR1 inflammation-responsive p150 (white histograms) and constitutively-expressed p110 (full histograms) isoform mRNA levels, compared to HPRT mRNA levels in primary patients. Histograms represent mean values for individual patient ± S.E.M (ctrl n=3; smoldering MM n=4; newly diagnosed MM n=4; relapsed MM n=7; PCL n=4). (e) ADAR1 relative expression in FPKM in primary CD138^{+} cells from patients (Stage I n=210, Stage II n=238, Stage III n=192); *p<0.05 value by unpaired, two-tailed Student's t-test. (f) Kaplan-Meier curves for overall survival (OS) of high (n=177) versus low ADAR1 (n=152) expressing cohorts in the CoMMpass study, stratified by International Staging System. p values by cumulative log-rank test.

Supplementary Figure 2, Related to Figure 2

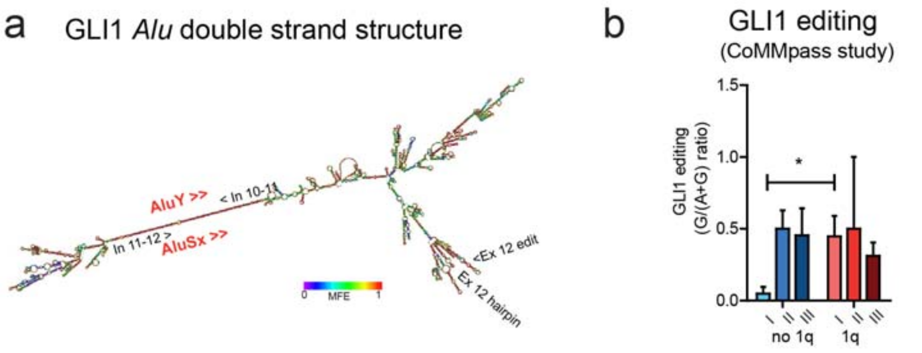

c Top 10 enriched gene sets in 1q amp

Gene Sets Enriched in 1q amp	Size	NES	NOM p-val	FDR q-val
Pathways in Cancer	320	1.54	0.001	0.08
MAPK Signaling Pathway	263	1.51	0.001	0.094
Signaling Pathways Regulating Pluripotency of Stem Cells	141	1.60	0.001	0.078
Insulin Signaling Pathway	135	1.65	0.001	0.079
Tight Junction	128	1.63	0.001	0.064
Leukocyte Transendothelial Migration	113	1.69	0.001	0.068
Hyperthropic Cardiomyopaty	83	1.61	0.001	0.071
Drug Metabolism Cytochrome P450	71	1.71	0.001	0.081
Pathogenic E. coli infection	55	1.76	0.001	0.05
Arginine and Proline Metabolism	52	1.83	0.001	0.037

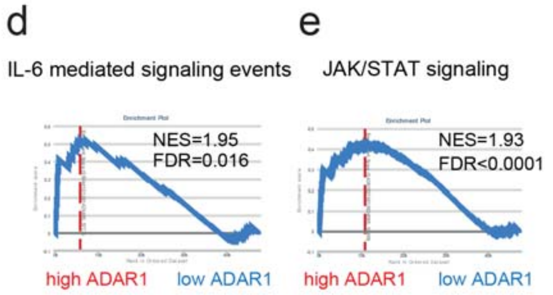

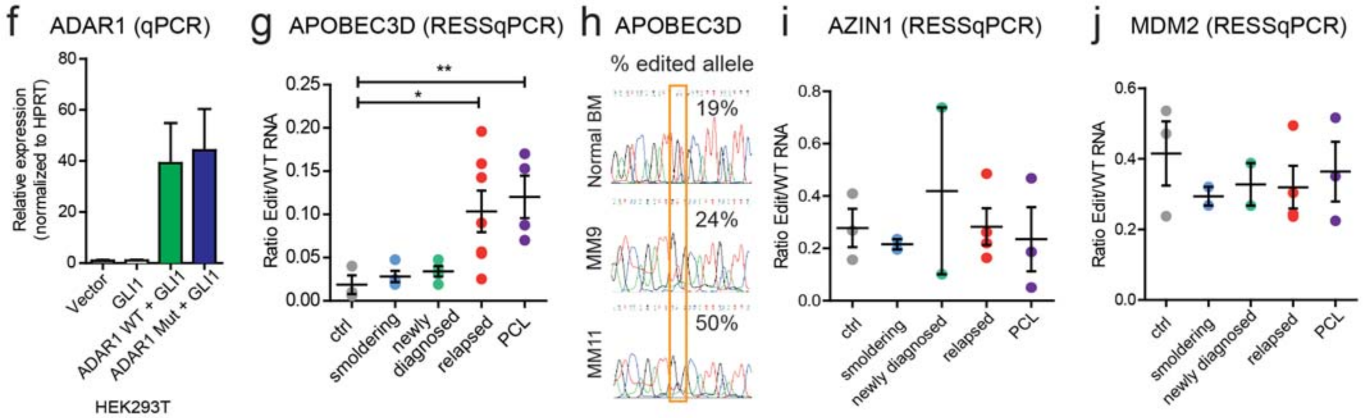

Supplementary Figure 2: (a) ViennaRNA predicted secondary structure formed by AluY and Alu Sx in GLI1 introns 10-11, and 11-12. (b) Proportion of editing events in GLI1 transcripts in samples with/ without 1q amplification, stratified by disease stage (Stage I n=7/7, Stage II n=2/6, Stage III n=5/4, respectively in 1q amp/ no 1q). *p<0.05 by unpaired, two tailed Student's t test. (c) Top 10 gene sets enriched in 1q amp patients. (d) Enrichment of IL-6 mediated signaling in high ADAR1 vs. low ADAR1 patients. (e) Enrichment of JAK/STAT signaling in high ADAR1 vs. low ADAR1 patients. (f) ADAR1 mRNA levels by qPCR after transient overexpression in HEK293T cells. Histograms represent mean ± S.E.M. of three independent experiments. (g) APOBEC3D editing in primary MM total MNCs by RESSqpCR. Dots represent the ratio of edit (G)/WT (A) APOBEC3D transcripts (mean values for individual patients ± S.E.M; ctrl n=3, smoldering MM n=4, newly diagnosed MM n=4, relapsed MM n=7, PCL n=4). *p<.05, **p<0.01, by unpaired, two tailed Student's t test. (h) Representative Sanger sequencing chromatograms for APOBEC3D; the yellow box highlights the double peak T/C (editing site in the antisense strand) in the transcripts, next to the percentage of edited mRNA/total transcripts, assessed as edit allele burden (%G/G+A). (i) AZIN1 editing in primary MM total MNCs by RESSqpCR. (mean values for individual patients ± S.E.M; ctrl n=3, smoldering MM n=2, newly diagnosed MM n=2, relapsed MM n=4, PCL n=3). (j) MDM2 editing in primary MM total MNCs by RESSqpCR (mean values for individual patients ± S.E.M; ctrl n=3, smoldering MM n=2, newly diagnosed MM n=2, relapsed MM n=4, PCL n=3).

Supplementary Figure 3, Related to Figure 3

a Summary of *in vivo* experiments

Sample	Tissue	Cell #	Cell transplanted	Route	Engrafted mice (by FC)	Immunophenotype
MM	BM	1.x10^6	Total BMNC	i.f.	0/13	NA
MM	BM	1.x10^6	CD138+ or CD138-	i.f.	0/9	NA
MM (MM3)	BM	1.x10^6	CD34-/CD138+ or CD34-/CD138-	i.f.	2/7	CD45dim
MM (MM3 serial)	BM,SP	1.x10^6	Human-enriched cells, pooled from BM and SP	i.f.	0/8	NA
MM (MM8)	BM	1.x10^6	Total BMNC	i.f.	3/10	CD45dim/CD319+/CD38+
PCL (MM9)	PCL	1.x10^6	Total BMNC	i.h.	7/7	CD45dim/CD319+/CD138+/CD38+
PCL (MM10)	PCL	1.x10^6	Total BMNC	i.h.	7/9	CD45dim/CD319+/CD138+/CD38+
PCL (MM9 serial)	BM, PC	2.x10^6	Pooled BM and PC cells	i.h.	6/6	CD45dim/CD319+/CD138+/CD38+

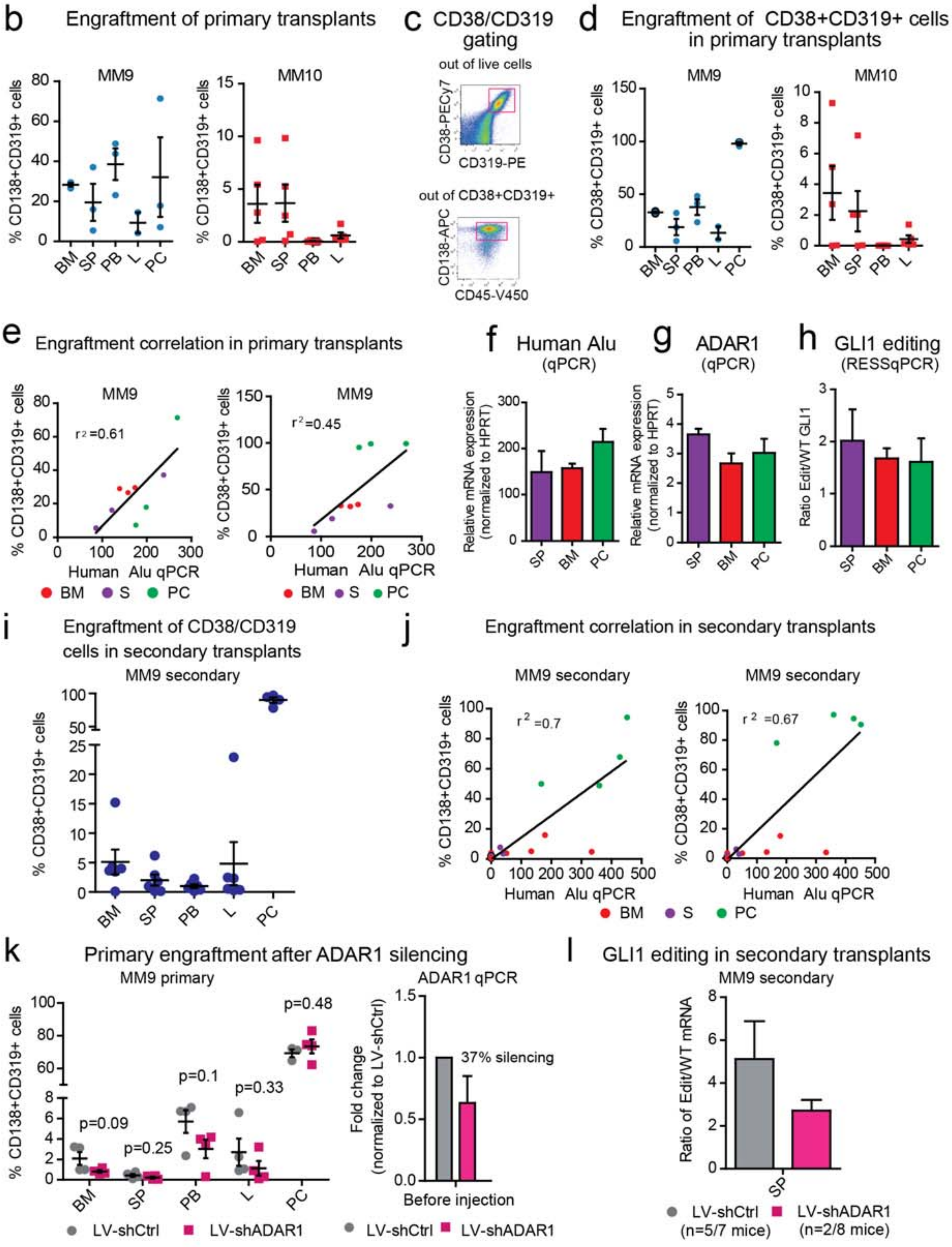

Supplementary Figure 3: (a) Summary of humanized MM mouse models established in immunocompromised animals. (b) Human CD138/CD319 double positive cell engraftment in primary recipients (MM9, n=3; MM10, n=5). (c) Representative dot plots of MM9-transplanted BM. (d) Human CD38/CD319 double positive cell engraftment in primary recipients (MM9, n=3; MM10, n=5). (e) Correlation of human RNA levels with engraftment, determined by flow cytometry analysis (% CD138/CD319 double positive cells left panel, %CD38/CD319 double positive cells right panel) in different tissues from primary recipients. (f) Human cell engraftment, determined by human-specific *Alu* qPCR in MM9 recipients (n=3 / tissue); (g) ADAR1 RNA expression levels by qPCR in MM9 recipients (n=3/tissue). (h) GLI1 editing by RESSqPCR in MM9 recipients (n=3/tissue). Histograms represent mean values ± SEM from individual mice in spleen (purple), bone marrow (red) and plasmacytomas (green). (i) Human CD38/CD319 double positive cell engraftment in secondary recipients (MM9 secondary, n=6). (j) Correlation of human RNA levels with engraftment, determined by flow cytometry analysis (left panel shows % of CD138/CD319 double positive cells, right panel shows % of CD38/CD319 double positive cells) in different tissues from secondary recipients (n=6). (k) Engraftment at end point of CD138/CD319 double positive cells in primary recipients of Lenti shADAR1/shCtrl-transduced MM9 cells (n=4 Lenti-shCtrl; n=4 Lenti-shADAR1). ADAR1 silencing was assessed by qPCR, before transplant, 48h post-lentiviral transduction, (l) GLI1 editing by RESSqPCR in MM9 secondary recipients after ADAR1 silencing (n=3 LV-shCtrl; n=2 LV-shADAR1). Histograms represent mean values ± SEM from individual mice in spleen tissues. p value by unpaired, two-tailed Student's t-test.

Supplementary Figure 4, Related to Figure 4

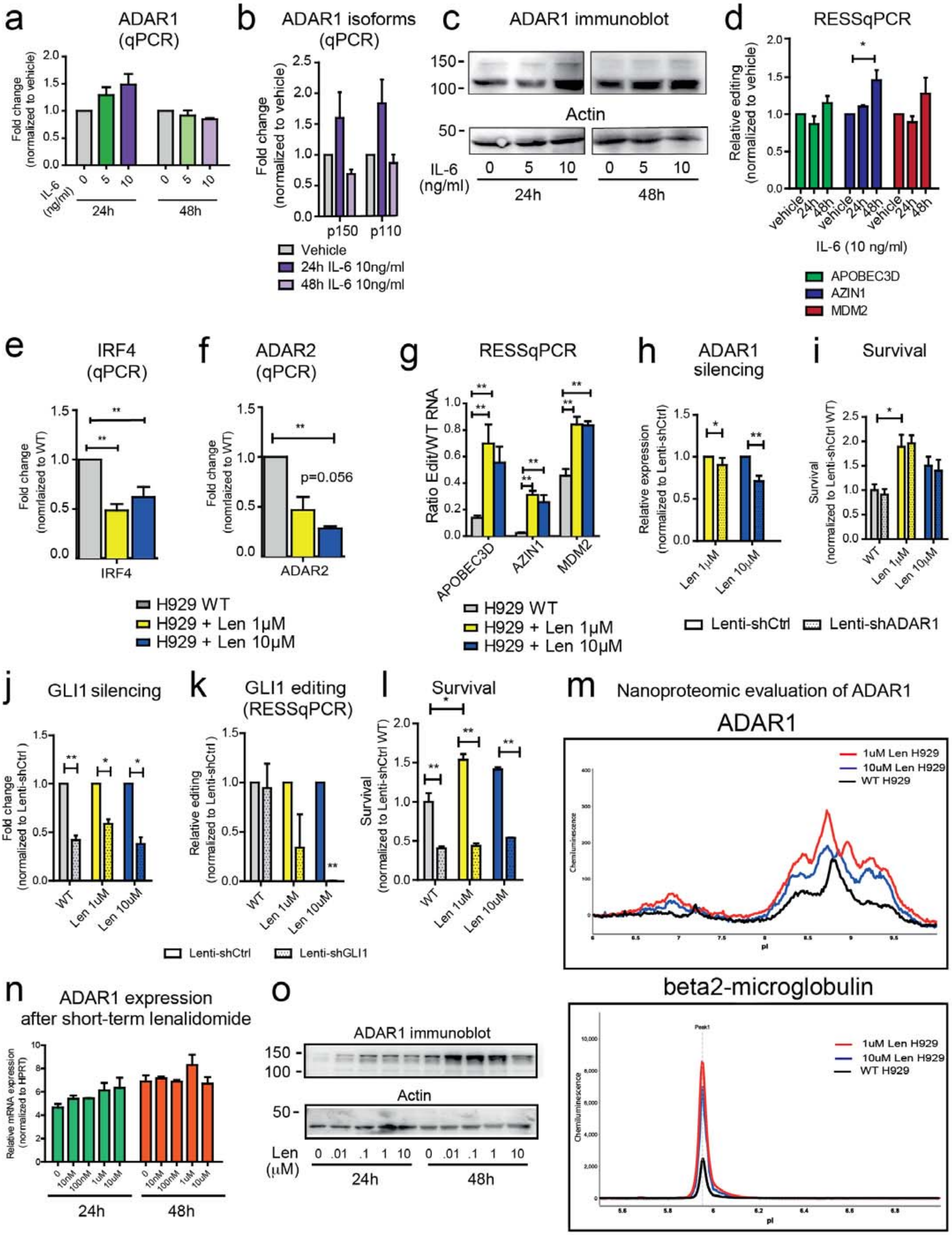

Supplementary Figure 4: (a) qPCR analysis of ADAR1 in H929 wild-type (WT) cells after IL-6 (0, 5, 10ng/ml) stimulation for 24-48 hours. (b) ADAR1 isoform expression by qPCR in vehicle-treated H929 WT cells or after IL-6 stimulation (10ng/mL). Histograms show mean ± SEM of three independent experiments. (c) Western blot analysis of H929 WT after IL-6 stimulation. Upper blot: polyclonal anti-ADAR1 antibody reveals bands at 150kDa and 110kDa corresponding to isoforms p150 and p110 in H929 cells after IL-6 stimulation (5-10ng/mL). Bottom blot: actin for normalization. (d) Evaluation of RNA editing by RESSqPCR in IL-6-stimulated cells after 24-48h, normalized to matched-vehicle. Histograms represent mean +/- SEM of at least three independent experiments; *p<0.05 by unpaired, two tailed Student's t test. (e) qPCR analysis of IRF4 mRNA (mean +/- SEM of at least three independent qPCR experiments) after prolonged lenalidomide treatment in H929 cells, compared to WT; **p<0.01 by unpaired, two tailed Student's t test. (f) qPCR analysis of ADAR2 mRNA (mean +/- SEM of at least three independent qPCR experiments) after prolonged lenalidomide treatment in H929 cells, compared to WT; *p<0.05 by unpaired, two tailed Student's t test. (g) Analysis of RNA editing of APOBEC3D, AZIN1 and MDM2 transcripts by RESSqPCR in lenalidomide-treated H929 cells, compared to WT; Histograms show mean +/- SEM of at least three independent qPCR experiments; **p<0.01by unpaired, two tailed Student's t test. (h) Survival of Len-resistant H929 cells upon lentivirally-enforced silencing of ADAR1 (mean ± SEM, n=3 per condition). *p<0.05 by by unpaired two tailed Student's t test. (h) ADAR1 mRNA levels by qPCR in lenalidomide-resistant cells, after silencing with Lenti-shCtrl/shADAR1, Histograms show mean ± SEM (n=3). *p<0.05, **p<0.01 by unpaired, two tailed Student's t-test. (i) Survival of Len-resistant versus WT cells after ADAR1 silencing (Lenti-shCtrl/Lenti-shADAR1. Histograms represent mean +/- SEM. *p<0.05, by unpaired, two tailed Student's t-test. (j) GLI1 transcript silencing in H929 WT versus Len-resistant cells, by lentiviral transduction with Lenti-shGLI1 or Lenti-shCtrl vectors, normalized to Lenti-shCtrl controls. Histograms represent mean +/- SEM values. *p<0.05, p<0.01 by unpaired, two tailed Student's t-test. (k) GLI1 editing by RESSqPCR in Len-resistant cells compared to WT after Lenti-shGLI1 transfection. **p<0.01 compared to Lenti-shCtrl by unpaired, two-tailed Student's t-test. (l) Survival of Len-resistant versus WT cells after GLI1 silencing (Lenti-shCtrl/Lenti-shGLI1. Histograms represent mean +/- SEM. (m) Representative nanoproteomic (CB1000) levels of ADAR1 in H929 WT or Len-resistant cells. β-2 microglobulin was used as internal normalization. All samples were loaded with equal protein amounts. (n) ADAR1 qPCR in H929 WT after short-term (24-48h) lenalidomide exposure (10nM-10uM). (o) Western blot analysis of H929 WT after short-term lenalidomide exposure. Upper blot: anti-ADAR1 monoclonal antibody reveals bands at 150kDa corresponding to isoforms p150 in H929. Bottom blot: actin for normalization.

Supplementary Figure 5, Related to Figure 4

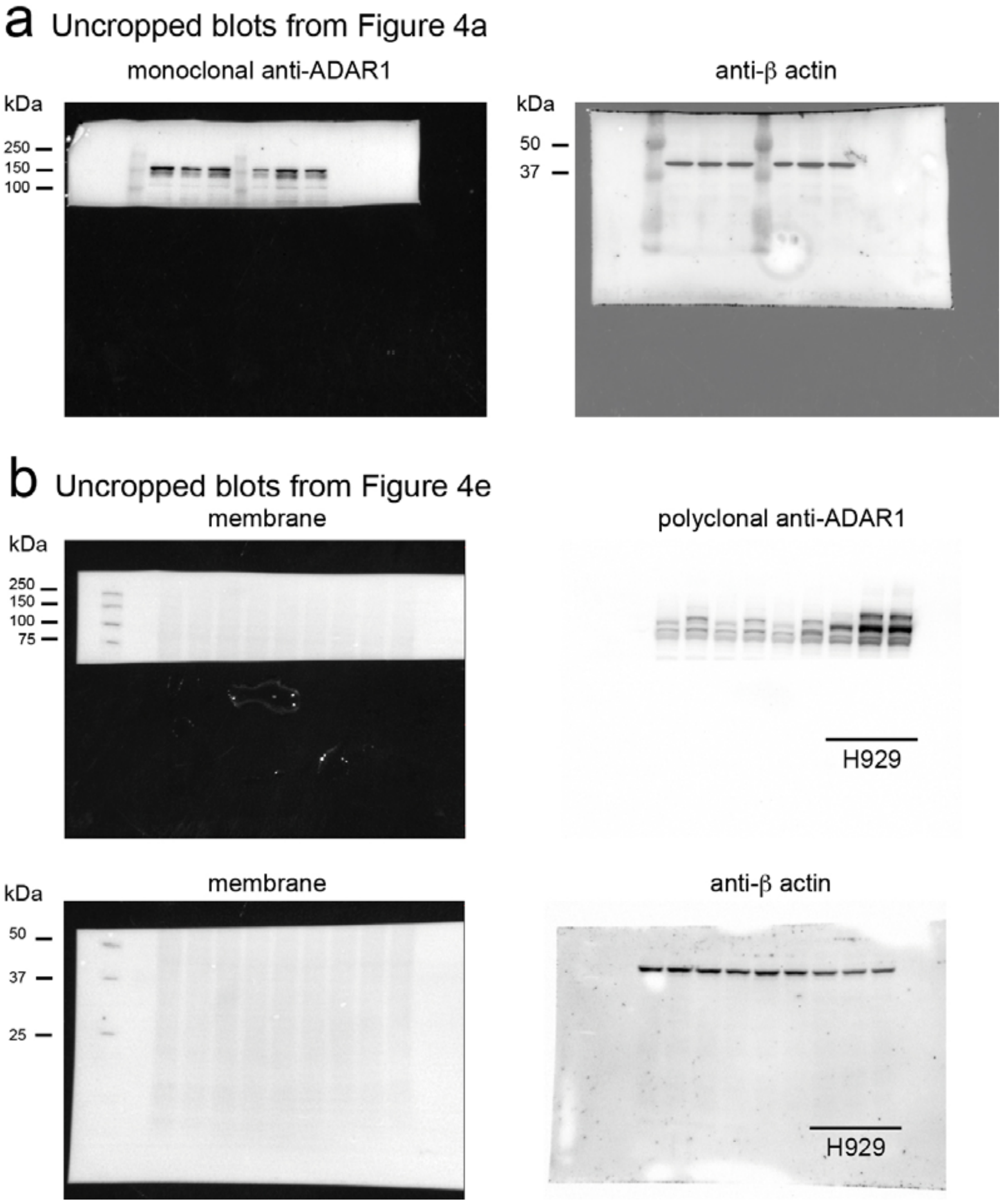

Supplementary Figure 5: (a) Uncropped blots from Western blot in Fig. 4a; (b) Uncropped blots from Western blot in Fig. 4e.

SUPPLEMENTARY METHODS

Cell lines and culture conditions

NCI-H929 (mycoplasma-free authenticated cell lines obtained from ATCC) were grown in RMPI-1640 Glutamax medium (Life Technologies) supplemented with 10% FBS and 0.05 mM β-mercaptoethanol. H929 cells were selected for this study because of their known amplification of chromosome 1q (containing at least 4 copies), and wild-type H929 cells were confirmed to have high endogenous levels of ADAR1 by qPCR. HEK293 (mycoplasma-free authenticated cell lines obtained from ATCC) were grown in DMEM medium (Life Technologies), supplemented with 10% FBS. All cell lines were maintained in T-25 or T-75 culture flasks and were cultured at dilutions of 1:3-1:6 every 2-4 days.

In IL-6 and lenalidomide short-term experiments, H929 wild type cells were plated at $3x10^5$ cells/ml, then exogenous IL-6 (R&D Technologies), or lenalidomide (AvaChem Scientific) was added directly to the culture media. Cells were collected for further analysis after 24 or 48 hours. To generate lenalidomide-resistant NCI-H929 cells, cells were treated twice a week with fresh lenalidomide at a final concentration of 1µM. Lenalidomide-treated and vehicle-treated control cells were sub-cultured for up to 10 weeks at the optimal concentration of $5x10^5$ cells/mL (log phase). Once the proliferation of cells was no longer inhibited by lenalidomide at 1 µM (10 weeks), as determined by Trypan Blue viability, lenalidomide treatment was scaled up to 10 µM[1]. All experiments were performed in triplicate.

Nucleic acid isolation, reverse transcription and quantitative PCR

Cell lines (0.5-2 x 10^6 cells) or primary purified MNCs (50-10x10^4 cells) were harvested in lysis buffer (Qiagen). RNA was purified using RNeasy extraction kits with a DNase (Qiagen) incubation step to digest any trace genomic DNA present. For RNA extraction from cell line lysates, samples were extracted using RNeasy mini columns, and for primary cells, samples were lysed and extracted using RNeasy micro columns. For standard qPCR analysis of relative mRNA expression levels, cDNA was synthesized using 50 ng - 1 µg of template RNA in 20-40 µL reaction volumes using the First-Strand SuperScript III Reverse Transcriptase Supermix (Life Technologies) followed by incubation with RNase H according to the manufacturer's protocol. All cDNA products were stored at -20°C.

Quantitative PCR was performed in duplicate in an iCycler (Bio-Rad) using SYBR GreenER Super Mix (Life Technologies). For standard qPCR, HPRT mRNA transcript levels were used to normalize Ct values obtained for each gene, and relative expression levels were calculated using the 2^{-ddCt} method. For all experiments, assays were repeated at least three times using separate RNA extracts and cDNA preparations using previously validated forward and reverse primers[2,3,4] or human-specific primers targeting *IRF4* (FW: 5'-GACATCTCAGACCCGTACAAAG-3', REV: 5'- GAAGGGTAAGGCGTTGTCAT-3').

High-fidelity PCR and Sanger sequencing analysis

For PCR and targeted Sanger sequencing analysis, 1-2 µL of first-strand cDNA templates were prepared for PCR using the high-fidelity KOD Hot Start DNA Polymerase kit according to the manufacturer's instructions (EMD Millipore). "Outer" primers used for sequencing produce predicted amplicons of approximately 150-250 nucleotides in length, and flank each editing site with approximately 50-100 bp on either side of the editing site to facilitate successful sequencing analysis. Production of amplicons of the predicted size was verified for each outer primer set by DNA gel electrophoresis using 10-20 µL of the reaction mixture separated on 2% agarose gels. Sanger sequencing was performed on ABI 3730xl DNA Sequencers (Eton Bioscience, San Diego, CA). Sequence chromatograms were analyzed using Chromas and peak heights calculated using ImageJ. Representative images of independent sequencing results (n=2 for each primary sample) are shown. For RNA editing analysis of sequencing chromatograms, ratios of edited/WT peaks were calculated using the raw peak amplitude of each sequence trace.

Flow cytometry analysis

Flow cytometry assays were performed using a BD LSRFortessa Cell Analyzer, BD FACSVerse™ System (BD Biosciences) and MACSQuant (Miltenyi). Data were analyzed using FlowJo (Treestar Inc.) software. Single cells derived from mouse tissues were stained with LIVE/DEAD fixable near-IR viability dye (Thermofisher) at 1:1000 in PBS. Samples were then washed and non-specific Fc receptor-mediated antibody binding was blocked by incubation with mouse and human FcR blocking reagent (BD Biosciences). Then samples were stained with anti-human CD138-APC (Miltenyi), CD319-PE (Miltenyi), CD38 PECy7 (BD Biosciences) and CD45 Brilliant Violet 450 (BD Biosciences). All incubations were performed at 4°C in dark. Human patient-

derived cells were gated as CD138+/CD319+ double positive cells, out of total single live cells, and then further gated according to the surface expression of CD38 and CD45. Representative dot plots of the gating strategy are shown in main and supplemental figures.

Colony formation assay
Lenalidomide treatment was withdrawn from culture for 5-7 days prior to colony assay initiation[5]. A total of 500 wild type or drug-resistant NCI-H929 cells were plated in methylcellulose (Miltenyi) in 12-well plates. Colonies (more than 40 cells) were scored after 14 days and serial replating assays were performed for an additional 10-14 days in culture. RT-qPCR on replated cells was performed on RNA extracted from 10 colonies.

***In vivo* bioluminescence imaging**
Mice were i.p. injected with luciferin (150mg/kg, Caliper Life Science) and anesthetized using isofluorane. Mice were imaged 6-8 minutes after i.p. injection. Images were acquired with a Xenogen IVIS-200 (PerkinElmer) after 1-5 minutes of exposure.

Measurement of serum immunoglobulin light chain levels
Peripheral blood was collected from each mouse by retro-orbital bleeding while animals were anesthetized with isofluorane. 10-50 μl of serum were used in enzyme linked immunosorbent assay (Human lambda or kappa ELISA kits, Bethyl Laboratories Inc.). The assay was carried out according to the manufacturer's guidelines.

Statistical analyses
The sample size of each experiment is limited by the availability of valuable samples specific for stage from patients. We use a definition of significance as a two sided alpha level of 0.05 and aim to have power of 0.80. Based on an expected effect size that is twice the standard deviation we can achieve 0.79 power with five samples per arm based on a normal distribution. The goal of each experiment is to get close to five or more samples per arm depending on clinical sample availability and viability. The effect size we are able to detect with this power is variable based on intra-arm sample variability (i.e. standard deviation).

For normally distributed data, unpaired two-tailed Student's t-tests were applied to determine differences in transcript expression, and values were expressed as individual data points or means (± SEM) from a minimum of two independent experiments. Mann-Whitney U test was used to compare the means of not normally distributed values (FPKM values derived from RNA seq). All statistical analyses were performed using Microsoft Excel or GraphPad Prism (San Diego, CA).

SUPPLEMENTARY REFERENCES

1. Lopez-Girona, A *et al.* Cereblon is a direct protein target for immunomodulatory and antiproliferative activities of lenalidomide and pomalidomide. *Leukemia* **26,** 2326–35 (2012).
2. Zipeto, M. A. *et al.* ADAR1 Activation Drives Leukemia Stem Cell Self- Renewal by Impairing Let-7 Biogenesis. *Cell Stem Cell* **19,** 1–15 (2016).
3. Jiang, Q. *et al.* ADAR1 promotes malignant progenitor reprogramming in chronic myeloid leukemia. *Proc. Natl. Acad. Sci.* **110,** 1041–6 (2013).
4. Crews, L. A. *et al.* An RNA editing fingerprint of cancer stem cell reprogramming. *J. Transl. Med.* **13,** 1–12 (2015).
5. Lopez-Girona, A. *et al.* Lenalidomide downregulates the cell survival factor, interferon regulatory factor-4, providing a potential mechanistic link for predicting response. *Br. J. Haematol.* **154,** 325–36 (2011).
